# Graphical Aids to the Estimation and Discrimination of Uncertain Numerical Data

**DOI:** 10.1371/journal.pone.0141271

**Published:** 2015-10-27

**Authors:** Myeong-Hun Jeong, Matt Duckham, Susanne Bleisch

**Affiliations:** 1 CyberGIS Center for Advanced Digital and Spatial Studies, University of Illinois at Urbana-Champaign, Urbana, IL, United States of America; 2 School of Mathematical and Geospatial Sciences, RMIT University, Melbourne, Victoria, Australia; 3 Institute of Geomatics Engineering, FHNW University of Applied Sciences and Arts Northwestern Switzerland, Muttenz, Switzerland; University of Akron, UNITED STATES

## Abstract

This research investigates the performance of graphical dot arrays designed to make discrimination of relative numerosity as effortless as possible at the same time as making absolute (quantitative) numerosity estimation as effortful as possible. Comparing regular, random, and hybrid (randomized regular) configurations of dots, the results indicate that both random and hybrid configurations reduce absolute numerosity estimation precision, when compared with regular dots arrays. However, discrimination of relative numerosity is significantly more accurate for hybrid dot arrays than for random dot arrays. Similarly, human subjects report significantly lower levels of subjective confidence in judgments when using hybrid dot configurations as compared with regular configurations; and significantly higher levels of subjective confidence as compared with random configurations. These results indicate that data graphics based on the hybrid, randomized-regular configurations of dots are well-suited to applications that require decisions to be based on numerical data in which the absolute quantities are less certain than the relative values. Examples of such applications include decision-making based on the outputs of empirically-based mathematical models, such as health-related policy decisions using data from predictive epidemiological models.

## Introduction

Across a variety of applications, human decisions are often based on numerical data where the absolute quantities are less certain than the relative values. For example, data output from predictive mathematical models, such as epidemiological models, frequently exhibit this feature. This research investigates the graphical representation of uncertain numerical data (such as the outputs from predictive epidemiological models) that makes relative (qualitative) judgments as effortless as possible, while making absolute (quantitative) estimation as effortful as possible. The aim of such graphics is to support human decision making under uncertainty, helping decision makers to have confidence in the relative values of data without giving these users spurious confidence in the absolute values.

Previous work has already used graphical representations of numerical data, in preference to presenting raw numbers, to improve its reliability for humans. For example, non-numeric images were used in [1] to improve system security in the context of the human limitations in remembering secure passwords. The study showed how recognition-based authentication (using images) could be more reliable and easier to use than traditional recall-based schemes [1]. In health and medical decision-making, a number of studies have used arrays of dots to help decision makers modify incorrect expectations about clinical management, and prevent difficulties in understanding health-related risks [2–4].

The task of estimating the number of dots in a graphical display is called *numerosity estimation*[5, 6]. A significant body of previous research has shown that humans exhibit systematic biases in numerosity estimation under different circumstances. For example, regular sets of dots have been shown to result in higher estimates of number than random sets, in all cases except the lowest numbers (which were judged accurately under both conditions) [5]. In general, arrays of dots tend to be underestimated when presented in dense clusters rather than spread out over a larger area [7, 8]. These findings have been explained by the occupancy model [6] and texture density [9]. Decreasing the distance between adjacent dots causes apparent overlapping of the area “occupied” by dots, leading to the underestimation of numerosity. In contrast, increasing the open space at the edges of dot arrays has been shown to lead to lower estimates of numerosity than arrays that fill the edges more completely [10]. Several other visual cues are implicated in human numerosity estimation, such as dot size [11]. Joining dots to make “bar-bells” also significantly reduces apparent numerosity under the same texture density [12]. Some evidence exists for a psychophysical basis for direct perception of numerosity in humans [13, 14]. For example, studies have identified neurons in both pre-frontal and parietal cortex that are tuned for numerosity [15, 16].

Related to the task of estimating absolute numerosity, the *relative* estimation of which of two graphics is more numerous is termed *numerosity discrimination*[10, 17]. Performance on numerosity discrimination tasks is strongly affected by the ratio between the two numbers being compared [17–19]. This ratio is captured by the Weber fraction, *k* = *J*/*N*, where *N* is the number of dots and *J* is a “just noticeable difference” (JND) between two stimuli [18]. For example, a Weber fraction of 0.10 indicates that a 10% change in magnitude of a stimulus (e.g., a 10% increase in the number of dots) can be reliably detected by subjects. Experiments have shown the Weber fraction for numerosity discrimination to be 0.162 in cases where *N* is between 8 to 30 [17]; 0.100 and 0.077 respectively for *N* = 25 and *N* = 100 [19]; and 0.168 and 0.155 respectively for *N* = 20 and *N* = 40 [6].

In summary, previous work has explored the extent to which different configurations of dots (e.g., regularity, clustering, more open space at the edge of a display) affect the accuracy of numerosity estimation and discrimination. However, the relationship between numerosity and discrimination for a particular configuration has not been investigated. Further, no work has yet investigated specifically the configurations best suited to achieve good performance at relative judgments in combination with poor performance at absolute estimation.

In addressing this gap in the previous literature, our central hypothesis is that hybrid randomized-regular arrays of dots are able to improve numerosity discrimination performance, when compared with random dot arrays, at the same time as decreasing numerosity estimation performance when compared with regular dot arrays. More specifically, our results show that:

- Both random and hybrid configurations reduce absolute numerosity estimation precision, when compared with regular dots arrays.
- Discrimination of relative numerosity is significantly more accurate for hybrid dot arrays than for random dot arrays.
- Human subjects report significantly lower levels of subjective confidence in judgments when using hybrid dot configurations when compared with regular configurations; and significantly higher levels of subjective confidence when compared with random configurations.

These results contribute to our understanding of how best to present numerical data in applications where decision makers may have confidence in relative judgments, but not in absolute data values. Our assumption is that effective decision-making using such uncertain numerical data can be better supported by representations that make relative judgments effortless at the same time as absolute judgments more effortful. These results contribute to our understanding of how best to present numerical data in such applications.

## Methods

This study compares three different types of non-numerical graphical representations of numerical data. Each representation consists of an array of dots. Our specific focus is to identify those representations that make the absolute numerosity estimation effortful, at the same time as making relative discrimination as effortless as possible.

### Participants

74 participants took part in the experiment. 33 participants were postgraduate students from Melbourne School of Engineering and Melbourne School of Population and Global Health at the University of Melbourne. 41 participants were students (28 undergraduates, 18 postgraduates) from Institute of Geomatics Engineering, FHNW University of Applied Sciences and Arts Northwestern Switzerland.

### Ethics statement

This study was conducted in October, 2014 and its conduct was approved by the University of Melbourne’s Human Research Ethics Committee (ID 1443039.1). Written informed consent was obtained from all subjects prior to participation.

## Materials

### Stimulus

The three different types of stimulus tested were:

I a regular array of dots distributed on a regular 10 × 10 grid;
II a randomized array of dots distributed on a regular 10 × 10 grid; and
III a randomized array of dots distributed randomly over the same area as the dots for type I and II arrays.

**Fig 1:**
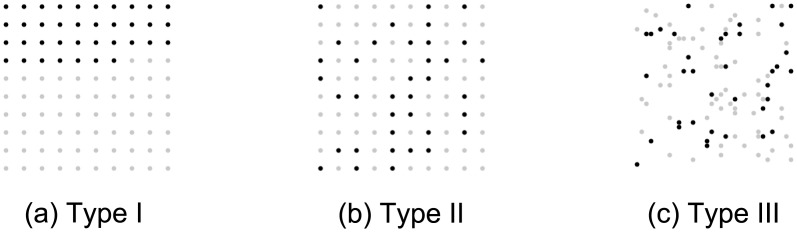
Examples of the three stimulus types for the number 37.

Examples of the three different types of stimulus are shown in Fig 1. The size of each stimulus was 300 × 300 pixels. The size of each dot was 7 pixels. The arrangement of dots depended on the stimulus type and numerosity. The stimuli were displayed on a computer screen with at least 1280 × 720 resolution and standard rotation at refresh rate of 60 Hz.

For the absolute numerosity estimation task, five different levels (numbers) were presented to subjects: 19, 37, 61, 73, or 91 dots. These levels were chosen to maximize comparability to previous research [5].

For the relative numerosity discrimination task, each of the same five (reference) numbers were compared with five or six other numbers, based on the Weber fraction. Table 1 shows the numbers of dots and the Weber fraction for the numerosity discrimination task.

**Table 1:** Trials for relative numerosity discrimination task. Numbers in parenthesis indicate the Weber fraction between reference and comparison numbers.

Reference	Comparison					
19	20 (0.05)	21 (0.10)	22 (0.15)	23 (0.21)	-	57 (2.00)
37	40 (0.08)	41 (0.10)	42 (0.14)	43 (0.16)	45 (0.21)	12 (2.08)
61	66 (0.08)	68 (0.11)	70 (0.14)	71 (0.16)	74 (0.21)	20 (2.05)
73	79 (0.08)	82 (0.12)	83 (0.14)	85 (0.16)	88 (0.21)	24 (2.04)
91	99 (0.08)	90 (0.10)	80 (0.14)	78 (0.16)	76 (0.20)	30 (2.03)

**Fig 2:**
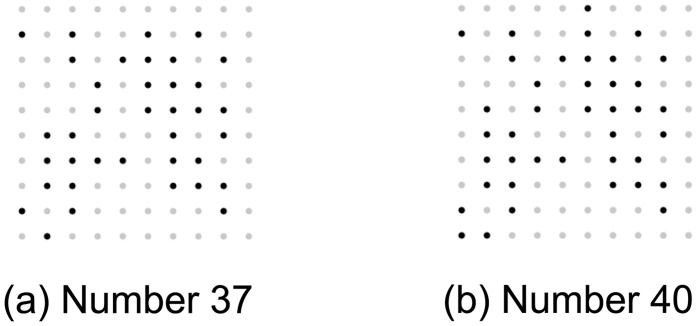
Example Type II stimulus for relative numerosity discrimination task (37 versus 40, Weber fraction 0.08).

The same types of stimulus were used in both absolute numerosity estimation and relative numerosity discrimination tasks. For stimulus Types II and III the dot locations were randomized across different stimulus instances. However, for the numerosity discrimination task, the lesser dot array contained a subset of the dots of the greater dot array for all Type II stimuli. Fig 2 shows an example of the Type II numerosity discrimination stimulus, with the less numerous array (Fig 2a) containing a subset of dots in the more numerous array (Fig 2b).

### Procedure

Two experiments examined the two related tasks: absolute numerosity estimation and relative numerosity discrimination. Each participant conducted the experiment using a customized web-based interface. Participants first completed the absolute numerosity estimation task and then conducted the relative numerosity estimation, consistent with [18]. Previous studies have shown that participants’ performance is not affected by reversing the order of these two tasks [10]. Thus, although we did not test performance for subjects performing the numerosity discrimination tasks before numerosity estimation, we would expect our results to be robust to reordering of these tasks.

**Fig 3:**
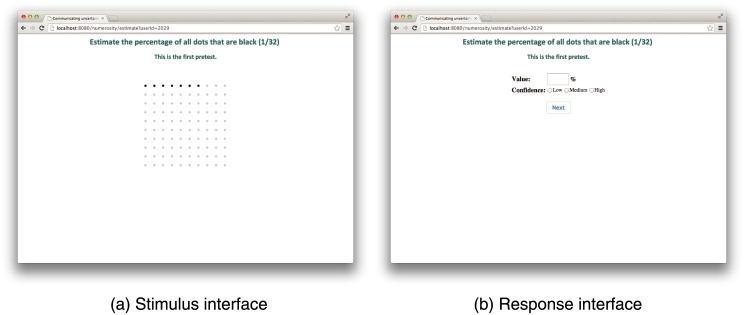
Example of stimulus (a) and response (b) interface for estimation task as presented to experimental subjects.

The numerosity estimation task required participants to estimate the number of black dots in a graphical dot array (see Fig 1). The instructions asked participants to estimate and not to count the number of dots present. Each array of dots was presented on screen only for a short time (2 seconds). This time was chosen, based on a pilot study, as a period long enough to minimize stress to the participants, but short enough to make counting of the dots by participants unlikely. After viewing each stimulus (see Fig 3a), participants were asked to estimate the number of black dots in the graphical dot array (see Fig 3b). Once a participant had entered his or her answer, using the computer keyboard, the next stimulus was presented to the participant.

Each participant was required to make estimates of 2 randomized repetitions of the 5 numbers across 3 stimulus types, leading to a total of 30 stimuli viewed by each participant (and a total of 148 estimates for each number across all participants). The order of stimulus presentation was randomized. An additional two pre-test stimuli were added to the beginning of each participant’s test, for which data was discarded. Participants were informed the first two stimuli were pre-tests and were for practice only.

**Fig 4:**
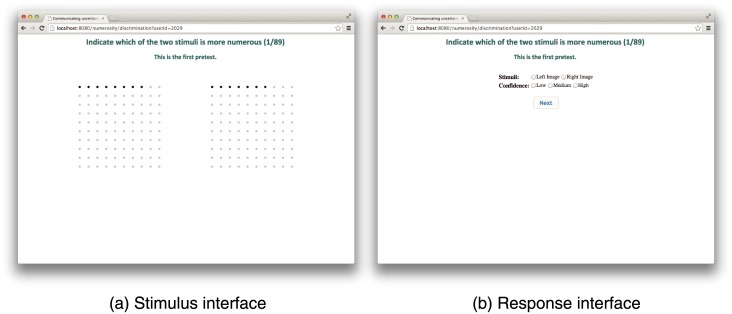
Example of stimulus (a) and response (b) interface for discrimination task as presented to experimental subjects.

The numerosity discrimination task presented pairs of dot arrays together. Participants were asked to judge which of the two graphical arrays of dots had the larger number of black dots. Numerosity discrimination stimuli were presented to participants for a slightly longer period (4 seconds) due to the relative difficulty of the task. After viewing each stimulus (Fig 4a), participants were asked to select the more numerous of the pair of dot array (Fig 4b). Once a participant had entered his or her answer (either the left or right dot array in the stimulus using the mouse) the next stimulus was presented to the participant. Each participant repeated this task in randomized order for the 29 pairs of numbers (Table 1) across the 3 stimulus types (a total of 87 stimuli per participant, and a grand total of 1332 estimates per reference number). The stimulus set was again preceded by 2 practice stimuli for which data was discarded.

Participants were also instructed to indicate their level of confidence in each answer—low, medium, or high—for both numerosity estimation and discrimination tasks. The level of confidence was reported after each stimulus via the web-based interface, at the same time as participants’ entered their absolute numerosity estimation or relative numerosity discrimination responses (see Figs 3b and 4b).

Data files containing the complete raw anonymized experimental data are available in the supporting information to this paper (S1 File).

## Results

### Absolute numerosity estimation

**Fig 5:**
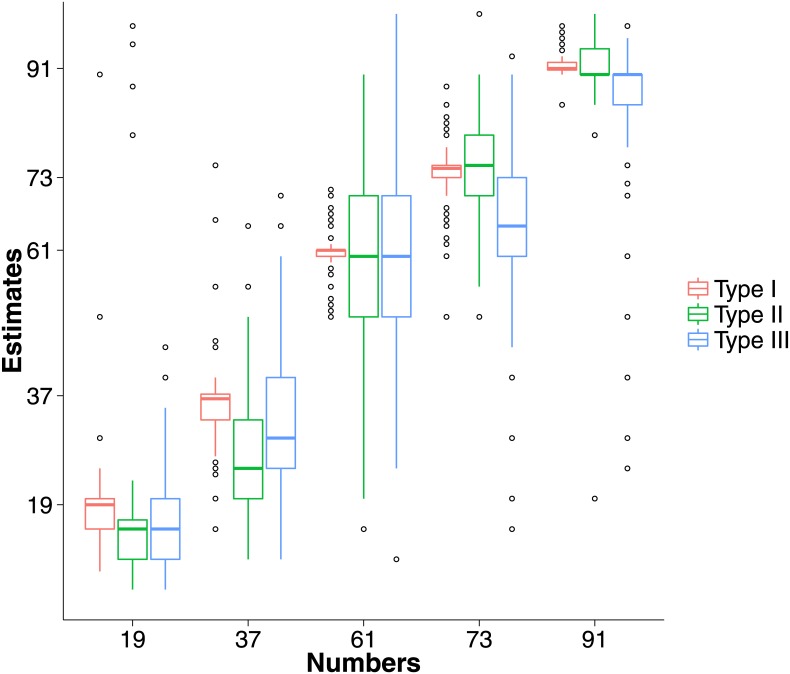
The spread of estimates based on type of stimulus.

A summary of participants’ absolute numerosity estimates is presented in Fig 5.

Estimates were compared with the true values across the five numbers using a bootstrapping M-estimator, due to the non-normal distribution of estimates. In addition, effect size was analyzed using Cliff’s delta [20], expressed in a standardized form using Cohen’s *d*.

As might be anticipated based on previous studies, the majority of tests exhibited a significant difference (*p* < 0.05) between the estimated and the true number (all cases except Type I, 19 and 91; Type II, 61 and 91; and Type III 61). Effect sizes were generally smaller for Type I than Type II and III stimuli.

However, it is immediately clear from Fig 5 that the variances of estimates for Type II and III stimuli are substantially larger than for Type I stimuli. This visual impression was confirmed statistically using Levene’s test, which indicated significant differences in variance at the 5% level between Type I versus Type II and Type I versus Type III estimates (i.e., all *p* < 0.01 except Type I and Type II for the number 19, *p* = 0.03). In contrast, there were no significant differences in variances for Type II and Type III stimuli for the numbers 19 (*F*(1,294) = 0.01, *p* = 0.93), 37 (*F*(1,294) = 3.90, *p* = 0.05), and 61 (*F*(1,294) = 0.26, *p* = 0.61).

**Fig 6:**
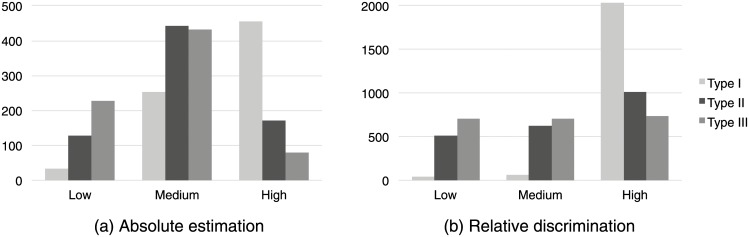
Counts of participants’ level of confidence in their judgments for a. numerosity estimation and b. numerosity discrimination tasks: The *x* axis presents the three stimulus types, grouped by confidence level: low, medium, or high. The *y* axis indicates the number of participants’ reporting that level of confidence in each class.

Lastly, participants’ confidence in their own responses was also analyzed. Fig 6a presents the histogram of participants’ confidence grouped confidence level across the three stimulus types.

Participants’ confidence was significantly affected by type of stimulus (Kruskal-Wallis test, *H*(2) = 495.9, *p* = 2.2 × 10^−16^). A post hoc test indicated all combinations of stimulus type differed significantly (i.e., significant differences at the 5% level between Type I versus Type II, Type I versus Type III, and Type II versus Type III, corrected for the number of tests).

Analyzing the correlation between participants’ confidence and the correctness of estimates showed a significant negative correlation between estimate correctness and participant confidence. As we might expect, participants’ confidence increases as the difference between participants’ estimates and real numbers decreases (Kendall’s tau, Type I stimulus *τ* = −0.28 and Type II stimulus *τ* = −0.23, *p* < 0.05). Type III stimuli also exhibited a significant difference between user confidence and estimate accuracy, but with a weaker correlation between the two (*τ* = −0.10, *p* = 0.0004).

### Relative numerosity discrimination

**Fig 7:**
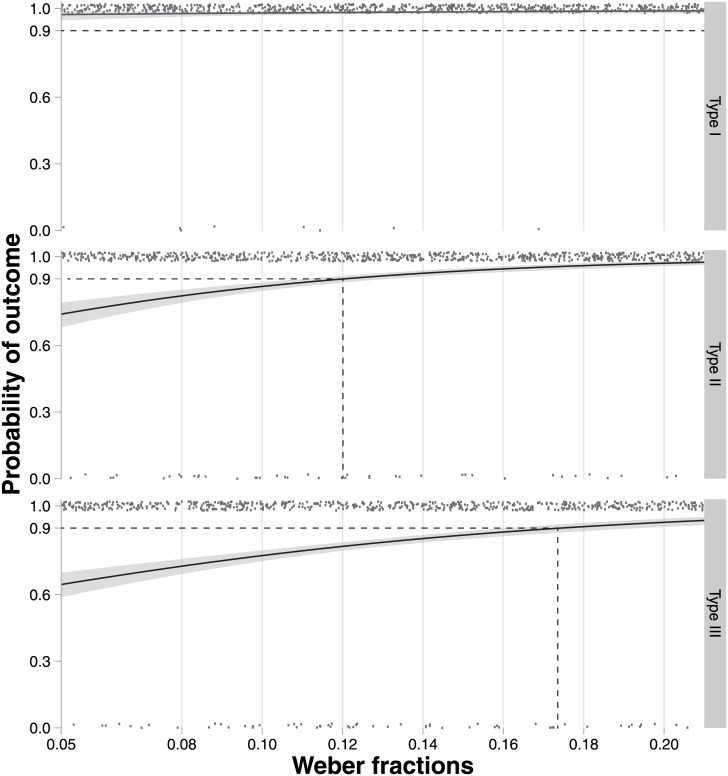
Relationship between Weber fraction and accuracy of participants’ judgments for numerosity discrimination. The abscissa of the 90% ordinate shows the Weber fraction corresponding to 90% accuracy of judgment by participants.

As would be expected from previous work, the correctness of participants’ judgments in the numerosity discrimination task is generally predicted by the Weber fraction (Fig 7). However, the precise relationship is strongly related to the stimulus type, with the Weber fractions required to achieve a particular accuracy increasing from Type I, to Type II, to Type III stimuli. For the Type I stimulus, participants always achieved over 90% accuracy, irrespective of Weber fraction. For Type II stimuli, 90% accuracy was achieved at a Weber fraction of above about 1.2; Type III stimuli achieved 90% accuracy at a Weber fraction of above about 1.75 (Fig 7 highlights the Weber fraction required to achieve 90% accuracy of judgment by participants for each stimulus type).

These visual impressions were confirmed using logistic regression analysis (Table 2). The analysis revealed statistically significant differences between all stimulus types. The odds ratios indicated that Type I outperformed Type II which in turn outperformed Type III.

**Table 2:** Model *β*-values and standard errors (SE), and odds ratios for logistic regression of numerosity discrimination task. ** indicates *p* < 0.01.

	*β* (SE)	Odds ratio, 95% CI
Type III vs Type I	2.29** (0.18)	9.85, CI(7.00 14.30)
Type III vs Type II	0.61** (0.10)	1.84, CI(1.51, 2.24)
Type II vs Type I	1.68** (0.19)	5.37, CI(3.76, 7.88)

The relationship between participants’ confidence and the correctness of judgments was again analyzed for the numerosity discrimination task (Fig 6b). Once again, stimulus type significantly affected participants’ confidence (Kruskal-Wallis test, *H*(2) = 1700, *p* < 0.01). Focused comparisons of the mean ranks between groups, corrected for the number of tests, indicated that all combinations of stimulus types exhibited differences significant at the 5% level.

Participants’ confidence was again significantly related to the accuracy of judgments for all stimulus types (Kendall’s tau analysis, *p* < 0.05). The relationship is also relatively highly correlated: *τ* = 0.22, *τ* = 0.26, and *τ* = 0.26 for Types I, II, and III stimuli respectively.

## Discussion

In line with previous work, the results of the absolute numerosity estimation task show that participants cannot, in general, estimate numerosity accurately, irrespective of type of stimulus. The exceptions were restricted to the numbers 19 (Type I), 61 (Types II and III), and 91 (Type I and Type II). This accuracy may in part be a result of using a frame around stimuli, making numbers closer to 0, 50, or 100 easier to estimate than, say, 37 or 73 [21]. Further, the clustering possible in the Type III stimulus is known to be associated with smaller estimates [7, 8].

However, the results show that the precision (variance) of participants’ estimates significantly decreases in moving from Type I (regular) to Type II (hybrid) or III (randomized) stimuli. Participants were also significantly less confident in their estimates when presented with random Type III or hybrid Type II stimuli when compared to regular Type I stimuli. Taken together, we can conclude from these results that Type II and III stimuli make the absolute numerosity estimation task harder, with participants having lower confidence in their performance.

Turning to relative numerosity discrimination, while participants performed best using a regular Type I stimulus, a hybrid Type II stimulus led to significantly better judgments than a randomized Type III stimulus. User confidence again reflects this pattern, with confidence in Type II stimuli lower than Type I but higher than Type III stimuli.

In summary, using the hybrid Type II stimulus led to worse numerosity estimation performance (in terms of precision of estimates) than for the regular Type I stimulus. The hybrid Type II stimulus performed no better than the randomized Type III stimulus for numerosity estimation in most cases. Conversely, numerosity discrimination using the hybrid Type II stimulus consistently outperformed the random Type III stimulus, even though it did not achieve the level of performance of the regular Type I stimulus.

Arguably, such characteristics—poor performance and confidence in absolute numerosity estimation combined with better performance and confidence in relative numerosity discrimination—make Type II dot arrays particularly well-suited to decision-making with numerical data where relative values are more certain than absolute values.

## Conclusions

This paper has investigated how differing arrangements of dots in graphical displays of numerical data can affect the accuracy of, and confidence in numerosity estimation and discrimination. In particular, the study focused on representations that help to make absolute numerosity estimates more effortful, while still facilitating effortless relative numerosity discrimination. The study found that irregular randomized arrays of dots arranged upon a regular grid led to: a. lower confidence in and poorer estimation of absolute numerosity than regular arrays of dots upon a regular grid; and b. higher confidence in and better relative discrimination between numerosity than randomized arrays of dots.

It seems reasonable to conclude, therefore, using the hybrid Type II stimulus for numerosity estimation is more effortful than using the regular Type I stimulus; and conversely that the hybrid Type II stimulus is less effortful than the random Type III stimulus for numerosity discrimination. Thus, using such hybrid arrays of dots represents a good compromise between facilitating relative discrimination and obscuring absolute estimation of numerosity.

These characteristics make Type II dot arrays particularly well-suited to decision-making scenarios with numerical data where relative values are more certain than absolute values. Examples of such scenarios include decisions based on the uncertain outputs of scientific models, such as epidemiological models. By more effectively communicating uncertainties to decision-makers in a form that does not inhibit decision-making itself should in turn lead to better decisions in these domains.

## Supporting Information

S1 File Metadata and data files containing anonymized raw experimental results in .csv format. (ZIP)
